# Absence of *kdr* resistance alleles in the Union of the Comoros, East Africa

**DOI:** 10.12688/f1000research.6567.1

**Published:** 2015-06-09

**Authors:** Yoosook Lee, Natalie Olson, Youki Yamasaki, Allison Chang, Clare Marsden, Ahmed Ouledi, Gregory Lanzaro, Anthony Cornel

**Affiliations:** 1 Vector Genetics Laboratory, Department of Pathology, Microbiology, and Immunology, School of Veterinary Medicine, University of California, Davis, CA, 95616, USA; 2 Department of Ecology and Evolutionary Biology, University of California, Los Angeles, CA, 90095, USA; 3 Université des Comores, rue de la Corniche, Moroni, Grande Comore, Comoros; 4 Department of Entomology and Nematology, University of California, Davis, CA, 95616, USA

**Keywords:** DIS, Dar es Salaam, CYP9K1, kncokdown resistence, insecticide, resistence

## Abstract

*Knockdown resistance *( *kdr*) and *CYP9K1* genotypes were detected by a MOLDI-TOF based SNP genotyping assay (Sequenom iPLEX) in samples of *Anopheles* *gambiae* collected at 13 sites throughout the Union of the Comoros and Dar es Salaam, Tanzania during February and March 2011. All *A*. *gambiae* specimens collected in the Comoros were homozygous for the susceptible *kdr *alleles (+/+) while 96% of *A. gambiae *from Dar es Salaam were homozygous for the East African *kdr *resistant genotype (E/E). In contrast, all specimens from Dar es Salaam and the Comoros were homozygous for the cyp3 allele (c3/c3) at the CYP9K1 locus; the locus has been implicated in metabolic resistance against pyrethroid insecticides in West Africa. All specimens had typical *A. gambiae *genotypes for SNPs within the *divergence Islands *on all three chromosomes. Although further spatial and temporal studies are needed, the distribution of *kdr *genotypes between the Comoros and Tanzania further supports isolation of the Comoros populations from *A. gambiae *populations on mainland Africa *.*

## Introduction

A majority of the human population residing in the Union of the Comoros (=94%) live in high malaria transmission zones ^1^. *Anopheles gambiae* and *Anopheles funestus* (Giles) are the major malaria vectors in the Comoros ^2^. Vector control efforts have concentrated on the adult stage using insecticide-treated bednets (ITNs) and indoor residual spraying (IRS) with DDT ^1^. ITN distribution was initiated in the Comoros in 2005 and by 2014 roughly 40% of the population has access to ITNs ^1^.

Limited insecticide resistance surveillance has been conducted on malaria vectors in Union of Comoros, with to date, published records stemming only from investigations in Mayotte (an island administered by France), where *A. gambiae* were susceptible to multiple insecticides except for a larvicide, temephos ^3^. Insecticide susceptibility studies have been conducted in neighboring East African countries such as in western Kenya (Chen *et al.* 2008. JME, Mathias *et al.* 2011, Malaria J, Ochomo *et al.* 2012 MVE), but little information is available on the coastal regions of Kenya. In Tanzania, information, based on small sample sizes, is available on the *kdr* allele frequency distribution in coastal districts of Muheza and Ilula (Dar es Salaam) ^4^ where about one third from Dar es Salaam were homozygous for the *kdr-*East (L1014S) mutation.

Here we present much needed data on *kdr* allele frequencies and include frequency data for a recently described pyrethroid metabolic resistance gene, *CYP9K1*. Allele frequencies for *Anopheles gambiae* collected at 13 sites in the Union of the Comoros, plus Dar es Salaam, Tanzania are presented ( Table 1).

**Table 1.** Sites, kdr, CYP9K1 information from *Anopheles gambiae* samples collected in the Comoros and Tanzania, February and March 2011. Numbers (#) indicate site locations on the map in Figure 1.

idx	Site	*Lat*	*Lng*	*Kdr* genotyped	+/+	E/+	E/E	%E	CYP9K1 genotyped	cyp3	%cyp3
1	Assimpao	-12.24	44.32	7	7			0	7	7	100
2	Boeninidi	-11.57	43.29	31	31			0	8	8	100
3	Bouni	-11.49	43.40	32	32			0	8	8	100
4	Fomboni	-12.28	43.73	28	28			0	5	5	100
5	Hoani	-12.26	43.67	20	20			0	8	8	100
6	Male	-11.89	43.51	17	17			0	14	14	100
7	Miringoni	-12.30	43.64	16	16			0	7	7	100
8	Moya	-12.31	44.44	68	68			0	8	8	100
9	Mutsamudu	-11.61	43.39	30	30			0	8	8	100
10	Ndremeani	-12.35	43.75	30	30			0	8	8	100
11	Ossivo	-11.59	43.28	18	18			0	8	8	100
12	Saliman	-11.68	43.27	4	4			0	4	4	100
13	Wala	-12.34	43.67	29	29			0	8	8	100
14	Wanani	-12.35	43.80	32	32			0	8	8	100
15	Dar es Salaam	-6.83	39.27	25		1	24	98	8	8	100
	**Grand Total**			**387**	**362**	**1**	**24**		**109**	**109**	

## Methods

**Figure 1.**
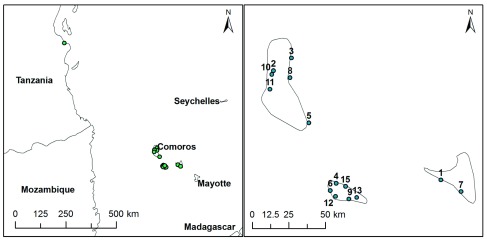
Collection sites in the Comoros and Tanzania. Site numbers corresponds to index number provided in Table 1.

A total of 362 indoor resting adults and larvae were collected from 13 locations from the three islands ( Figure 1) making up the Union of the Comoros between February and March, 2011. Larvae were individually rinsed twice in bottled mineral water and placed in 80% ethanol for downstream genomic DNA extraction. A collection of *A. gambiae* sensu lato from Furvela, Mozambique were collected using light traps inside houses. Mosquitoes from Dar es Salaam were obtained from Dr. Kija Ngh’abi at Ifakara Health Institute.

Samples were transported to the UC Davis Vector Genetics Laboratory for further genetic assay. DNA was extracted using a DNeasy extraction kit (Qiagen, Valencia, CA). Species were determined based on the combination of species diagnostic assays ^5, 6^ and a divergence island SNP (DIS) genotyping assay ^7^.

For *DIS*, *kdr* and *CYP9K1* genotyping, we used the Sequenom iPLEX Gold Genotyping Reagent Set (Catalog number: Sequenom 10158) on a MassArray (Sequenom) mass spectrometer at the UC Davis Veterinary Genetics Laboratory. This assay was slightly modified from the original DIS assay ^7^ by adding the *kdr* and *CYP9K1* markers, as described in Supplemental Document S1.

## Results & discussion

*A. gambiae* from Dar es Salaam, Tanzania, had the *kdr-*East (L1014S) ** genotype at a frequency of 96%, which is higher than the frequency previously reported from Dar es Salaam by Kabula *et al.* ^7^ where respectively, 1/3 and 2/3 of their samples were homozygous and susceptible for *kdr-*East (L1014S). In contrast, all *A. gambiae* from the Comoros were homozygous for the susceptible *kdr* alleles. All *A. gambiae* from both Tanzania and the Comoros were homozygous for the cyp3 allele for the CYP9K1 gene. All specimens from Furvela, Mozambique were *A. merus* (30/35) or *A. arabiensis* (5/35) and were excluded from further analysis.

Significant pressure to select for resistance to pyrethroid insecticides in *A. gambiae* and other indoor biting and resting malaria vectors likely occurs throughout sub-Saharan Africa because of intense IRS and ITN usage. A recent study in Mali noted an adaptive introgression of *kdr* resistant alleles from *A. gambiae* stably incorporated into the *A. coluzzii* genome under high ITN coverage environments ^8^. A similar genomic signature of adaptive introgression was also observed in Ghana ^9^. *A. gambiae* populations in the Comoros have had the opportunity, via transport by boat or air, to acquire resistant *A. gambiae* genotypes from neighboring countries such as Tanzania where high levels of insecticide resistance have been reported ^10^. The failure of the Comoros population to acquire insecticide resistance alleles despite long term exposure to insecticide pressure ^1^ may potentially be due to several factors or combination of factor including: (1) ITN coverage (<25% compared to >60% Mali) is not high enough to drive selection for resistance, (2) these populations are very isolated from mainland populations, requiring them to develop resistance *de novo* rather than from gene flow from neighboring populations, and/or (3) *A. gambiae* on the Comoros may be exophilic.

Our study provides much needed information regarding the genetics of insecticide resistance in *A. gambiae* populations in the Comoros Islands. Although the malaria vectors in Comoros appear to be genetically predisposed to insecticide susceptibility, it is possible that these mosquitoes have developed phenotypic resistance via alternative mechanisms such as metabolic resistance other than *CYP9K1* or behavior resistance (e.g. exophily). Further studies are needed to establish levels of phenotypic resistance against insecticides, as well as bionomics of the malaria vectors in this region to understand the impact of insecticide-based malaria control measures in the Comoros.

## Supplementary materials

**Supplemental Document S1.**

Modification of the original DIS assay in 7.

## References

[1] WHO: World Malaria Report 2014. Switzerland: World Health Organization 2014.2014 Reference Source

[2] Ayala D Goff GL Robert V: Population structure of the malaria vector *Anopheles funestus* (Diptera: Culicidae) in Madagascar and Comoros. *Acta Trop.* 2006;97(3):292–300. 10.1016/j.actatropica.2005.12.002 16464433

[3] Pocquet N Darriet F Zumbo B: Insecticide resistance in disease vectors from Mayotte: an opportunity for integrated vector management. *Parasit Vectors.* 2014;7:299. 10.1186/1756-3305-7-299 24984704 PMC4094441

[4] Kabula B Kisinza W Tungu P: Co-occurrence and distribution of East (L1014S) and West (L1014F) African knock-down resistance in *Anopheles gambiae sensu lato* population of Tanzania. *Trop Med Int Health.* 2014;19(3):331–341. 10.1111/tmi.12248 24386946 PMC4190685

[5] Scott JA Brogdon WG Collins FH: Identification of single specimens of the *Anopheles gambiae* complex by the polymerase chain reaction. *Am J Trop Med Hyg.* 1993;49(4):520–529. 8214283 10.4269/ajtmh.1993.49.520

[6] Favia G Lanfrancotti A Spanos L: Molecular characterization of ribosomal DNA polymorphisms discriminating among chromosomal forms of *Anopheles gambiae* s.s. *Insect Mol Biol.* 2001;10(1):19–23. 10.1046/j.1365-2583.2001.00236.x 11240633

[7] Lee Y Marsden CD Nieman C: A new multiplex SNP genotyping assay for detecting hybridization and introgression between the M and S molecular forms of *Anopheles gambiae.* *Mol Ecol Resour.* 2014;14(2):297–305. 10.1111/1755-0998.12181 24119184 PMC3947471

[8] Norris LC Main BJ Lee Y: Adaptive introgression in an African malaria mosquito coincident with the increased usage of insecticide-treated bed nets. *Proc Natl Acad Sci U S A.* 2015;112(3):815–820. 10.1073/pnas.1418892112 25561525 PMC4311837

[9] Clarkson CS Weetman D Essandoh J: Adaptive introgression between *Anopheles* sibling species eliminates a major genomic island but not reproductive isolation. *Nat Commun.* 2014;5:4248. 10.1038/ncomms5248 24963649 PMC4086683

[10] Kabula B Tungu P Malima R: Distribution and spread of pyrethroid and DDT resistance among the *Anopheles gambiae* complex in Tanzania. *Med Vet Entomol.* 2014;28(3):244–252. 10.1111/mve.12036 24192019 PMC10884793

