# Supplementary material for: Absence of kdr resistance alleles in the Union of the Comoros, East Africa
Source: F1000Res. 2015 Jun 9;4:146. [Version 1] doi: 10.12688/f1000research.6567.1 (PMC4544377; doi:10.12688/f1000research.6567.1)
Supplement: Peer review history

# Peer review: Referee response for version 1

**DOI:** 10.5256/f1000research.7052.r9761

**Published:** 2015-08-03

**Authors:** Frédéric Simard, Carlo Costantini

**Affiliations:** 1 Maladies Infectieuses et Vecteurs : Ecologie, Génétique, Evolution et Contrôle, Institut de recherche pour le développement, Université Montpellier, Montpellier, France

The paper by Lee et al. provides strong evidence for the absence of mutant alleles at the well-characterized *kdr *locus in populations of the major malaria mosquito *Anopheles gambiae* from three islands of the Comoros archipelago. The lack of *kdr* mutants in these populations contrasts with the high frequency of the East-African *kdr* mutation (L1014S) in a continental population from Dar es Salam, Tanzania. These findings are consistent with results of work carried out by our research group in the neighbouring island of Mayotte, showing no evidence for phenotypic insecticide resistance in *An. gambiae, *as well as the absence of target site mutations at the *kdr *locus on this island. The authors conclude that, altogether, these results suggest restricted gene flow between continental and island populations of *An. gambiae *in this area.

The paper is concise and clear. The title and abstract are appropriate, and they reflect adequately the content of the paper.

There are, however, a few minor shortcomings to be addressed:

- The number and position of the sampling sites are not consistent in the text, Table and Figure: 13 sampling sites are mentioned in the text and abstract, 15 are shown in the Table and Figure; there are also inconsistencies with the identification codes between the table and the figure, and the caption of Table 1 indicates these codes with the symbol ‘#’ whereas the corresponding column name in the body of the Table is ‘idx’.
- In the first sentence of the ‘Results & discussion’ section, the authors state that “ *A. gambiae *from Dar es Salaam, Tanzania, had the *kdr*-East (L1014S) genotype at a frequency of 96%,…”. The sentence should either state that 96% of the specimens were homozygous for the *kdr *allele (as in the abstract) or that the *kdr *mutation (instead of “genotype”—it is more appropriate as this is a single nucleotide polymorphism) was found at a frequency of 98% (as shown in Table 1).
- It is mentioned in the Introduction that “population access to ITNs” in 2014 in the Comoros was 40%, whereas it is reported that “ITN coverage” is <25% when discussing the results. Please explain the difference between these figures.
- Table S1 in the Supplementary Materials still uses the non-Linnean nomenclature of molecular forms instead of *An. gambiae* and *An. coluzzii*; we think this should now be superseded by the Linnean taxonomic nomenclature.
- There is little information in the paper about the CYP9K1 locus; for example, does it confer cross-resistance to DDT? Is the cyp3 allele wild-type or ‘resistant’? What is the phenotype of ‘resistant’ alleles? One or more references would be useful in this respect.
- In which year were the mosquitoes sampled in Dar es Salam?

 

Moreover, the authors should:

- Include the references ‘Chen et al. 2008. JME, Mathias et al. 2011, MalariaJ and Ochomo et a l. 2012 MVE’ which are cited in the text (‘Introduction’) in the reference list;
- Specify in the ‘Methods’ (or perhaps in Table 1) which of the 362 specimens were collected as resting adults and which ones were collected as larvae; also, which steps were taken to avoid sampling individuals coming from the same mosquito progeny in larval samples, as this could have an impact on observed genetic diversity.
- Correct the reference number for the Kabula *et al. *paper cited in the ‘Results & discussion’ section.
- Use the abbreviation “ *An.*” rather than “ *A.*” for *Anopheles*, in agreement with taxonomic conventions and community usage.
- Italicize the adverb “ *sensu lato*” given that it is from the Latin.

Finally, it is in our view inappropriate to infer about gene flow between the Comoros and the African continent based on these results. (i) The locus is under selection, which is not ideal for gene flow inference, and, as discussed by the authors, the nature, strength, and geographic distribution of selective pressures may differ between the Comoros and the single continental population that was sampled. (ii) The level of resistance to pyrethroids and other insecticide compounds due to the L1014S mutation is probably low in *An. gambiae *( Ranson *et al.*, 2000), and may differ according to genetic background. (iii) All other genetic markers used in the study, including CYP9K1 argue for gene flow occurring between these populations.

Because there are no phenotypic data for the level of resistance of the *An. gambiae *populations that were included in the study, and because no historical and accurate data on insecticide usage in the Archipelago are available, it is at this point rather speculative to explain the absence of *kdr *mutations in the Comoros populations.

Accordingly, the selective pressures for insecticide resistance operating on these populations need to be assessed before inferring the dynamics of gene exchange between these island and continental populations. As acknowledged by the authors, vector behaviour (feeding preferences, feeding time, endo-exophagy, endo-exophily) also needs to be investigated before any conclusion can be put forward to explain the differences observed.

We have read this submission. We believe that we have an appropriate level of expertise to confirm that it is of an acceptable scientific standard.

# Peer review: Referee response for version 1

**DOI:** 10.5256/f1000research.7052.r8980

**Published:** 2015-08-03

**Authors:** Frederic Tripet

**Affiliations:** 1 Centre for Applied Entomology and Parasitology, School of Life Sciences, Keele University, Staffordshire, UK

This is an interesting short report describing the prevalence of two loci incriminated in metabolic and target-site resistance to pyrethroid pesticides in populations of the malaria mosquito *A. gambiae* s.s. on the Comoros Islands. The cyp3 allele of the CYP9K1 gene was found in all island specimens and also in a single population sampled from mainland Tanzania. In contrast the East African *kdr *(L1014S) allele was not found on the islands suggesting that these populations are fully geographically isolated from mainland populations, or alternatively that pesticide selection pressures are not very high on the islands due to limited vector control programmes. I just have a few general suggestions that may help make the current report a little bit more informative in key areas and thus make it more relevant to a broader readership.

The current introduction and discussion are very succinct...

Are these 2 loci the only major loci involved in resistance to pyrethroids and why were they chosen in particular? Additional background with references would be useful in the intro.

As noted by the authors, there is limited information on pesticide resistance in general on the Comoros. The authors also state: 'Our study provides much needed information regarding the genetics of insecticide resistance in *A. gambiae* populations in the Comoros Islands.' It looks to me that what is first and foremost really needed are detailed bioassay surveys of pesticide resistance for the island populations. These would have provided a better context for the current study and would have made the interpretation of the distribution on the two *kdr* resistance loci easier. Is the cyp3 allele here an ancestral allele or has it swept through due to selection?

Given the above, the main selling point of this study lies, in my view, in the use of the *kdr* locus as a marker for introgression as done previously in studies of West African *A. gambiae* populations. For many years, *kdr* was the only marker suggesting an absence of gene flow between *A. coluzzii* and *A. gambiae *s.s. in Mali. Hence, I would suggest expanding that part of the discussion a little bit. The current section omits some interesting parallels and details.

In the same line of thought, discussing the new findings with those generated using neutral markers in a previous study for the same islands ( Marsden *et al*., 2013) would be useful, again because of the similarities with approaches (neutral versus non-neutral markers) used in past studies of reproductive isolation.

The authors mention possible gene flow from the mainland via boats. It may be worth mentioning that the main boat connections are from Mayotte, Zanzibar and Madagascar whose *A. gambiae* populations are not included in this and previous study. Why is that? What is the status of those populations? If the Comoros islands were to be used for possible mosquito release programmes, re-colonization from neighbouring islands would possibly also be a concern. On the other hand, gene flow from the Mozambique coast can only be dismissed if additional sampling was made from that region.

This is clearly an area of the world whose vector populations are greatly understudied. Given that these islands represent some of the best locations for testing mosquito release programmes, this study, albeit a small contribution, represents an important step in the right direction.

I have read this submission. I believe that I have an appropriate level of expertise to confirm that it is of an acceptable scientific standard.

# Peer review: Referee response for version 1

**DOI:** 10.5256/f1000research.7052.r8978

**Published:** 2015-07-22

**Authors:** Beniamino Caputo, Verena Pichler

**Affiliations:** 1 Department of Public Health & Infectious Diseases, Sapienza University of Rome, Rome, Italy

**Title**

Please specify which kdr has been genotyped.

**Abstract**

The abstract provides an adequate summary of the article.

Please insert that you collected just “1” site in Tanzania at Dar es Salaam. Moreover, you have 14 sampling sites in the Comoros according to Table 1.

Please specify that you refer to *Anopheles gambiae* (Coetzee et al., 2013).

**Introduction**

Please explain your interest on CYP9K1 gene and add citations.

Add references on insecticide resistance surveillance in Mozambique if any are available.

Add the objective of the study (gene-flow of continental versus island).

Minor comments:

In the 1st paragraph, the last sentence change “has” to “had”.

In the second paragraph substitute citation with numbers.

**Methods**

Study design and methods are quite well explained.

Please specify how many larvae and adults have been collected from 13 locations from the three islands, and give more details in the results section.

Modify Figure 1 according to Table 1 (Figure 1: image on the right side: you have missed out the number 14 and the number 15 is in a different location to that stated in Table 1. Therefore the number 15 in Figure 1 should be changed to a 14 and number 15 should be added at Dar es Salaam, Tanzania).

In the table you list 15 collection sites but here you cite only 14.

**Results**

Please specify the statement “malaria vectors in Comoros appear to be genetically predisposed to insecticide susceptibility”and add more details and references.

Correct the citation Kabula *et al.* ^7 ^to Kabula *et al.* ^4^

The samples in Mozambique seem not related to the study since only *A. merus* or *A. arabiensis* have been found.

The discussions are balanced and justified based on the results obtained, even if continental sample (Tanzania) is very small.

We have read this submission. We believe that we have an appropriate level of expertise to confirm that it is of an acceptable scientific standard, however we have significant reservations, as outlined above.
